# Hydroclimate and vegetation variability of high Andean ecosystems

**DOI:** 10.3389/fpls.2022.1067096

**Published:** 2023-01-20

**Authors:** Julieta Carilla, Ezequiel Aráoz, Javier Foguet, Elvira Casagranda, Stephan Halloy, Alfredo Grau

**Affiliations:** ^1^ Instituto de Ecología Regional (IER), Universidad Nacional de Tucumán (UNT)- Consejo Nacional de Investigaciones Científicas y Técnicas (CONICET), Tucumán, Argentina; ^2^ Animal and Plant Health Directorate, Biosecurity, Ministry for Primary Insdustries, New Zealand, Ministry for Primary Industries, Wellington, New Zealand; ^3^ Facultad de Ciencias Naturales e Instituto Miguel Lillo, UNT, Tucumán, Argentina

**Keywords:** Cumbres Calchaquíes, ENSO, lake area, NDSI, remote sensing indicators, SAVI, snow-ice cover, vegetation index

## Abstract

Mountain ecosystems are sensitive to climate fluctuations; however, the scarcity of instrumental data makes necessary the use of complementary information to study the effect of climate change on these systems. Remote sensing permits studying the dynamics of vegetation productivity and wetlands in response to climate variability at different scales. In this study we identified the main climate variables that control vegetation dynamics and water balance in Cumbres Calchaquíes, NW Argentina. For this, we built annual time series from 1986 to 2019 of Soil Adjusted Vegetation Index (SAVI, to quantify spare vegetation productivity), lake area, and snow-ice cover of peatlands, as indicators of mountain productivity and hydrology. We used a decompose function to explore trend, seasonality and random signal of the three-time series, and explored for significant changes in the mean value of consecutive periods. We used correlational analysis to explore their associations with climate records at local, regional, and global scales. The results showed that, SAVI and hydrological indicators presented different fluctuation patterns more pronounced since 2012, when they showed divergent trends with increasing SAVI and decreasing lake area and snow-ice cover. The three indicators responded differently to climate; SAVI increased in warmer years and lake area reflected the water balance of previous years. Snow-ice cover of peatlands was highly correlated with lake area. La Niña had a positive effect on lake area and snow-ice cover and a negative on SAVI, while El Niño had a negative effect on SAVI. Fluctuations of lake areas were synchronized with lake area in the nearby Argentinian puna, suggesting that climate signals have regional extent. The information provided by the three hydroclimate indicators is complementary and reflects different climate components and processes; biological processes (SAVI), physical processes (snow ice cover) and their combination (lake area). This study provides a systematic accessible replicable tool for mountain eco-hydrology long-term monitoring.

## 1 Introduction

High Andean ecosystems are particularly sensitive to climate change, because they are adapted to extreme environmental conditions such as low temperatures, high evapotranspiration and low CO_2_ pressure, which are affected by climate change in steep gradients (Pauli and Halloy, 2019). Morales et al. (2015) showed an aridization trend in the last decades in the dry Andes of NW Argentina. At the same time, Vuille et al. (2018) showed faster rates of warming in higher elevation than lowlands, which together with the increasing impact of anthropogenic pressure (Izquierdo et al., 2018), may threaten the vegetation adapted to these Andean ecosystems. Due to the importance of the Andes in terms of biodiversity, water supply to lowland cities and agriculture (Olson et al., 2001; Anderson et al., 2021), and particularly, the dry Central Andes includes lithium triangle, so the anthropic pressure is likely to increase in the next few years (Izquierdo et al., 2015a) there is an urgent need to understand their dynamics and the main drivers of changes. However, the scarcity of climate records in these environments fosters the exploration of alternative hydroclimate indicators to gather long-term information.

Climate is a complex system that results from the interaction of global circulation, regional processes, and local topography. Global atmospheric circulation influences the annual and interannual variability of mountain climate (Garreaud, 2009; Espinoza et al., 2020; Arias et al., 2021). In the Central Andes, the South American Monsoon System is responsible for precipitation seasonality through moisture transportation from tropical to subtropical latitudes during austral summers (Garreaud et al., 2003; Marengo et al., 2012; Kock et al., 2019). El Niño Southern Oscillation (ENSO), the coupled atmospheric-oceanic, has differential effects worldwide (Glantz et al., 1991). Some authors evidenced a strong association between ENSO and subtropical high Andean ecosystems (Halloy, 2002; Morales et al., 2015), reflected not only in water availability but also in vegetation growth (Carilla et al., 2018a; Maggi et al., 2020). Other authors reported the influence of other large-scale circulation forces, such as the Pacific decadal oscillation (PDO) and the Atlantic Multidecadal Oscillation (AMO), on Andean precipitations (Morales et al., 2015; Kayano et al., 2019).

The dry Central High Andean ecosystems (c. 18 to 35 LS; Tovar et al., 2022) present extreme climatic conditions, with life growing at its physiological limits mainly due to low temperature and limiting resources such as water, nutrients, CO2 and O2 (Körner, 1999; Pauli and Halloy, 2019). In these systems, biological diversity and vegetation productivity are mainly concentrated in wetlands, which play a central role in wildlife and water regulation, as well as human occupation and pastoralism history (Izquierdo et al., 2018). In arid and semi-arid systems, water balance, which results from the difference between precipitation inflows and evapotranspiration outflows, is an important indicator of water availability for the ecosystem functioning (Stephenson, 1990; Stephenson, 1998). On the other hand, green indices, such as NDVI is widely used to quantify vegetation productivity (e.g. Xue and Su, 2017), which is known to be influenced by water balance, the dominant community, soil types and topography (Carilla et al., 2013; Morales et al., 2015; Casagranda et al., 2019). However, very little is known about the interaction between proximate and distant drivers that modulate Andean climate and vegetation dynamics.

Remote sensing has revolutionized our understanding of ecosystems functioning at broad scales because they allow systematic and spatialized surveys through large areas. The spectral information facilitates the characterization of different biophysical patterns and processes, such as land cover (Olson et al., 2001), spatial distribution of vegetation classes (Carilla et al., 2018b; Oyarzabal et al., 2018), or the description of wetland dynamics (Izquierdo et al., 2015b). Most remote sensing studies use indices as the main tool for monitoring Earth cover (e.g. NDVI, is widely used to infer vegetation functioning; Xue and Su, 2017). Besides, the systematic data acquisition of remote sensors facilitates the implementation of long-term studies, encompassing multiple spatial scales, which are useful to discriminate between temporal trends and high frequency variability (Perez et al., 2010). The use of remote sensed products to estimate ecosystem productivity or hydrological balance has been the focus of some studies in the Andes (Carilla et al., 2013; Morales et al., 2015; Bianchi et al., 2017; Casagranda et al., 2019; Maggi et al., 2020). However, proxies can be quite dependent on the climatic, environmental and topographic characteristics of each particular system, so the calibration with known ground processes is recommended to ensure that their interpretation is accurate.

The goal of this study is to identify the climate variables that control water balance and vegetation dynamics in Cumbres Calchaquíes (NW Tucumán, Argentina). For this, we characterized the long term hydrological and vegetation dynamics, through three hydroclimate indicators derived from satellite images and analyzed their association with climate records. We combined Landsat 5 Thematic Mapper (TM), Landsat 7 Enhanced Thematic Mapper Plus (ETM+), and Landsat 8 Operational Land Imager (OLI) images to build a 30-year time series of remotely sensed data on vegetation productivity (through Soil Adjusted Vegetation Index, SAVI), lake area, and snow-ice cover of peatlands in Cumbres Calchaquies. We assessed the association of these indicators with different climate variables and with the dynamics of water area of lakes located in north and central Argentinian Andes (to test the capability of these indicators to reflect regional weather patterns). We hypothesized that undergoing gradual changes in mountain productivity and hydrology in Cumbres Calchaquíes are reflected in these indicators.

## 2 Materials and methods

### 2.1 Study area

**Figure 1:**
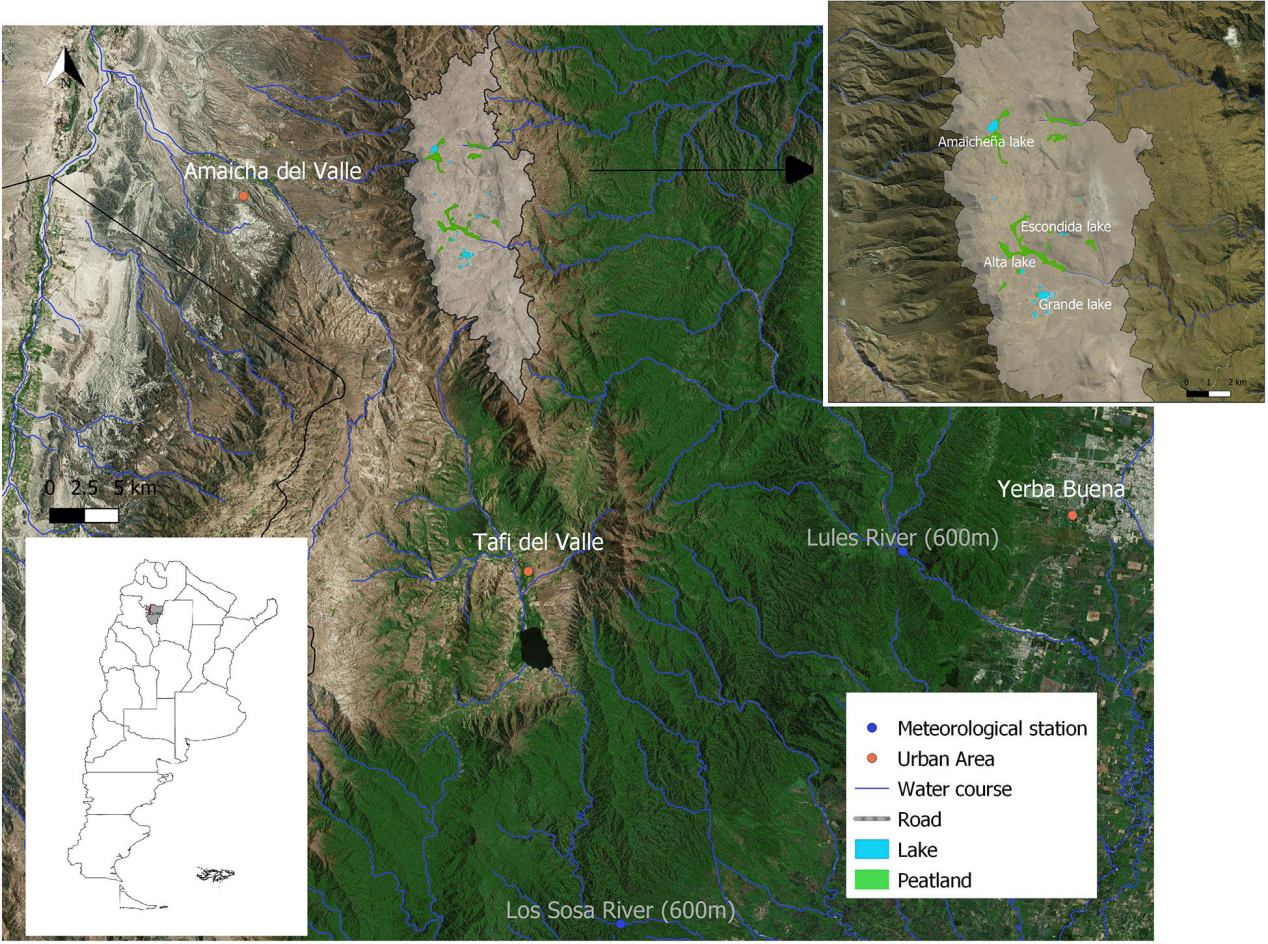
Map of the study area in Cumbres Calchaquíes mountain range, with Huaca Huasi lakes and peatlands located over 4000 masl indicated with the gray polygon (right corner), in Tucuman province, NW Argentina (left corner). The main cities are indicated with an orange point and the two meteorological stations at lower elevation with blue points.

Cumbres Calchaquíes is located in the homonymous Provincial Park, in Tucumán province, NW Argentina (-26S, -65W). Geologically, the area belongs to the Pampean system, with Precambrian metamorphic bedrock (Halloy, 1985). The Huaca Huasi high plateau is located over 4200 m asl (c. 150 km2; **Figure 1**), with dozens of temporary and permanent glacial lakes, surrounded by peaks around 4700 m asl. The area presents an annual rainfall of 300 mm concentrated in the summer months (December to April) and a mean annual temperature of c. 3°C (records from 2015-2017) with high solar radiation throughout the year and strong winds occurring mainly during winter (Halloy et al., 2020). This mountain is the source of streams that feed rivers downstream, important for human use in both slopes; eastern (subtropical montane forest; e.g. high basin of Salí – Dulce hydrological system) and western (dry valleys; Yocavil river system) slopes (García et al., 2017).

The area corresponds to the High Andean biogeographic province (Cabrera, 1976; Carilla et al., 2018). Calchaquí district (Halloy, 1985) characterized by high levels of plant diversity and limited distribution ranges (Halloy et al., 2020). Vegetation communities are dominated by Iro grasslands (*Festuca ortophylla* Pilg*.)*, in hillsides and plains, and cushions of Azorella compacta Phil. in rocky hillsides. Diverse cushion plants communities (e.g. *Adesmia crassicaulis* Phil*., Pycnophyllum convexum* Griseb.) are dominant in plains with silty-clayey and stony soils (with higher cover than the iro grasslands). Peatlands (locally known as bofedales or vegas), a particular southern America cushion bog are dominated by Juncaceae and Cyperaceae cushions plants (e.g. *Oxychloe andina* Phil., *Distichia muscoides* Nees & Meyen; Halloy et al., 2020). The local fauna is represented by the native camelid *Lama guanicoe* Müller (guanaco), the big rodent *Lagidium viscacia* Molina (chinchillón), the carnivorous *Puma concolor* Linnaeus (Puma), the birds *Vultur gryphus* Linnaeus (Condor Andino), *Chloephaga melanoptera* Eyton (Guayata), among others. The main impact on vegetation is herbivory by native and exotic grazers (cattle and donkeys are present up to 4000 m asl), mountaineering to a lesser extent, and off-road vehicles in particular areas (Lomáscolo et al., 2014).

### 2.2 Image processing and data collection

Image processing and data collection were done using Google Earth Engine, a cloud-based platform that allows free access and processing of a wide variety of geospatial data (Gorelick et al., 2017). We used three Landsat collections provided by the U.S Geological Survey (USGS): “USGS Landsat 8 Surface Reflectance Tier 1” (2013 - 2020), “USGS Landsat 7 Surface Reflectance Tier 1” (2000 - 2019), and “USGS Landsat 5 Surface Reflectance Tier 1” (1986 - 2012). These products are already transformed to surface reflectance in an effort of the USGS to provide useful data to conduct historical studies of land surface change. Landsat 5 and 7 were corrected using The Landsat Ecosystem Disturbance Adaptive Processing System (LEDAPS) that follows the 6S (second simulation of the satellite signal in the solar spectrum) radiative transfer model (Masek et al., 2006), while Landsat 8 collection images were corrected using the LaSRC algorithm as described in (Vermote et al., 2016). In order to obtain a historical series of cloud-free images, we used the bit information referred to clouds and clouds shadows in the “pixel_qa” band and created a binary cloud mask for each scene to remove unwanted pixels. We obtained 1901 scenes available from 1986 to January 2020, with a spatial resolution of 30 x 30 m (i.e. each pixel has 900 m^2^). The study area intersects four Landsat Paths & Rows: 231/78, 231/79, 230/78, 230/79. Due to its height, the study area remains frequently free of clouds even in the wet season.

We built two-time series of median mosaics grouping images by calendar year (n=33) and by quarter (n=132); each quarter corresponds roughly to the four seasons; quarter 1: January, February, March -JFM- (summer), quarter 2: April, May, June -AMJ- (autumn), quarter 3: July, August, September -JAS- (winter), quarter 4: October, November, December -OND- (spring), for the southern hemisphere.

We classified three types of environments that are dominated by different vegetation communities: peatlands, plains, and hillsides. Peatlands (410 ha were classified as peatlands) are known to be temporarily flooded environments with highly green productive vegetation communities (Izquierdo et al., 2015b; Meneses et al., 2020; Anderson et al., 2021), distinguishable from the semiarid matrix. Plains (1099 ha) and hillsides (3736 ha) are dominated by different vegetation communities and their soils have different water retention capacities. We classified hillside environments whenever the slope of a pixel, estimated from a 30 m digital elevation model, was above 10%.

#### 2.2.1 Soil-adjusted vegetation index (SAVI)

SAVI is useful to quantify spare vegetation productivity, minimizing the brightness effect of soil on vegetation reflectance (Gilabert et al., 2002; Cao et al., 2014), and is calculated as:

(NIR – RED)/(NIR + RED + L) * (1+L)

Where, NIR (near-infrared) and RED are two bands of the spectrum, and L (soil adjustment factor) = 0.5, used for intermediate vegetation cover (Huete, 1988). We applied a correction of the SAVI series since 2013 when L8 began to function. We statistically quantified differences in the values for the overlapping years from L5-L7 (2000 - 2012) and L7-L8 (2014- 2019). The vegetation signal of L8 was systematically higher than the other sensors by 0.0337 (p<0.05) thus we corrected L8 based SAVI by subtracting this value.

For both, the annual and the quarterly series, we extracted the median of SAVI values for the area above 4000 m asl. The median is a conservative indicator of vegetation productivity that is not affected by extreme values. Thus, we obtained eight SAVI indicators; annual and quarterly median SAVI for the study area, and annual SAVI for the three classified types of environments (peatlands, plains and hillsides).

#### 2.2.2 Water area of lakes

We selected four lakes located in the area; Grande (a group of several shallow interconnected lakes), Amaicheña, Alta, and Escondida lakes (**Figure 1**), as indicator of water balance (Song et al., 2014). These lakes differ in the morphology of their watersheds. Grande, Amaicheña and Alta are ‘playa’ lakes (Hutchinson, 1967). They are set in sedimentary basins surrounded by low, rounded hills, with a slope of about 3%. In contrast, Escondida is a glacial valley lake in a narrow steep-sided valley. Slope steepness is 10 times higher, increasing to 38%. As a result, the first three have a high variability of lake area. We obtained the annual maximum water area of the four lakes from the dataset JRC Monthly Water History, V1.1. This dataset contains accurate monthly maps of water surface classification around the world from Landsat 5, 7, and 8 satellites, from 1986 to 2018 (32 years; Pekel et al., 2016). We calculated the average water surface of the four lakes for each year and the four quarters of every year. Thus, we obtained five indicators of lake areas; annual mean area of each lake and annual average of the four lakes, previously normalized so all the lakes had similar weight in the average. We previously tested for correlations (Spearman) between the four lakes’ annual series.

#### 2.2.3 Snow-ice cover of peatlands

With the same Landsat dataset, we estimated three annual measures of snow-ice cover in a group of 10 peatlands (as indicators of winter characteristics, such as intensity, precipitation, cloudiness, and wind; Kim et al., 2015), covering 212 ha (from 410 ha classified as peatlands), using the normalized snow index (NDSI; Hall & Riggs, 2011) calculated as:

(GREEN - SWIR)/(GREEN+ SWIR)

Where GREEN and SWIR (short wave infrared) are two bands of the spectrum. We calculated the annual mean and maximum area of snow-ice cover of peatlands by counting pixels with NDSI greater than zero and multiplying by 0.09 in order to express the area in hectares c. Finally, we estimated the duration, in months, of the snow-ice period identifying the first and last month with more than one snow-ice cover pixel.

Thus, we obtained three snow-ice cover of peatlands indicators; mean, maximum, and duration (**Figure 1**; the same peatlands used for SAVI calculation).

### 2.3 Climate records and indicators

Long-term climate instrumental records are scarce in this mountain range. Thus, we used 18 climate variables combining different sources. Climate variables encompass three spatial scales that are likely to influence the ecohydrology of the focal system: local, regional and global. Local climate records included data gathered in the same study area in the context of ongoing long-term research and data from meteorological stations in the surrounding middle and lowlands. We used soil temperature records from data loggers located at three elevations (4040, 4450, and 4740 m asl) within the study area (data from GLORIA network, Carilla et al., 2018). We used records from each data logger separately and we estimated their mean value, which is likely to be more stable and conservative. We gathered temperature, precipitation, and river gauge records from two meteorological stations at lower elevations that are likely to capture variation in precipitation of the upper watershed (**Figure 1**). For the regional scale, we used temperature, precipitation, and vapor pressure from the Climate Research Unit (CRU), from the high-resolution dataset (0.5 latitude by 0.5° longitude grid; Harris et al., 2020). For the global scale, we explored six indices capturing the large-scale circulation forces related to the Pacific and Atlantic oceans: SOI, SST, AMO, TSA, PDO, and MEI (**Table 1**).

**Table 1:** Description of the climate variables used, spatial and temporal scales and source.

Spatial Scale	Description	Climate variables and codes	Temporal scale	Source
Local instrumental records	Data loggers installed in Cumbres Calchaquíes, at -10 cm of depth at three elevations: 4040, 4450 and 4740 masl.	Soil temperature T_4040 T_4450 T_4740	2009-2015; 2017-2020	Carilla et al., 2018a
	Meteorological stations located in Los Sosa river, at 600m asl,	Air temperature T_LSosa	1993-2018	https://snih.hidricosargentina.gob.ar/Filtros.aspx#
		Precipitation P_LSosa	2010-2020	
		River record R_LSosa	1993-2018	
	Meteorological stations located in Potrero de las Tablas, at 600 masl river	Air temperature T_Potrero	1987-2018	https://snih.hidricosargentina.gob.ar/Filtros.aspx#
		Precipitation P_Potrero	1987-2018	
		River record R_Potrero	1987-2018	
Regional variables	Interpolated from meteorological stations from the Climate Research Units database (CRU TS 4.04 grid-box data for 26.75 S, 65.75 W) dataset on a 0.5° latitude by 0.5° longitude grid	Air temperature T_CRU	1985-2019	Harris et al., 2020 https://rdcu.be/b3nUI
		Precipitation P_CRU	1985-2019	
		Vapor pressure V_CRU	1985-2019	
Large scale climate variables	Mean annual monthly Southern Oscillation Index standardized (measure of atmospheric pressure used as a proxy of precipitation)	SOI	1985-2019	https://www.bom.gov.au/climate/current/soihtm1.shtm
	Mean annual Sea Surface Temperature (East Central Tropical Pacific Ocean N3.4 region).	SST	1985-2019	https://www.cpc.ncep.noaa.gov/data/indices/
	Atlantic multidecadal oscillation (An index of surface temperature from the North Atlantic temperature - 5x5 degree resolution)	AMO	1985-2019	https://psl.noaa.gov/data/climateindices/list/ https://www.psl.noaa.gov/data/timeseries/AMO/
	Tropical Southern Atlantic Index (anomaly of the average of monthly sea surface temperature from 20S and 10E – 30W)	TSA	1985-2019	https://psl.noaa.gov/data/climateindices/list/ https://www.psl.noaa.gov/data/climateindices/list/
	Pacific decadal oscillation index (standardized principal components of temperature series of surface water in the Pacific Ocean, North 20°N).	PDO	1985-2019	https://psl.noaa.gov/data/climateindices/list/ https://www.psl.noaa.gov/data/correlation/pdo.data
	Bi-monthly Multivariate ENSO index (encompass sea level pressure, SST, surface wind and longwave radiation) over the tropical Pacific basin (30°S-30°N and 100°E-70°W)	MEI V2	1985-2019	https://psl.noaa.gov/data/climateindices/list/ https://psl.noaa.gov/enso/mei/data/meiv2.data

We used data of El Niño and La Niña events, although, they are gradual fluctuations over several years. We identified eight years of El Niño events: 1986-87 (moderate), 1987-88 (strong), 1991-92 (strong), 1994-95 (moderate), 1997-98 (very strong), 2002-03 (moderate), 2009-10 (moderate), 2015-16 (very strong) and seven of La Niña Events: 1988-89 (strong), 1995-96 (moderate), 1998-99 (strong), 1999-00 (strong), 2007-08 (strong), 2010-11 (strong), 2011-12 (moderate). (from: http://www.bom.gov.au/climate/enso/outlook/#tabs=ENSO-Outlook-history).

We used data from annual lake area of 15 lakes located along the Argentinean Puna (high Andean desert, north and central Argentinean Andes, from 1986 to 2017 period), from Casagranda et al. (2019), to assess the spatial scope of Cumbres Calchaquíes response to a regional climate signal. These lakes were separated into two groups according to relatively synchronous variations in their water surface, resulting in two geographical zones: NE and SW. The general trend in both lake zones is a decrease in water surface, although they show great individual variability.

### 2.4 Data analysis

#### 2.4.1 Characterization of the three indicators temporal series

We used a decompose function to explore the trend, seasonality and random signal of the time series of annual SAVI, average lake area, and snow-ice cover of peatlands. We explored for significant changes in the mean value of consecutive periods (dating breaks) of the trend component (breakpoints function, Zeileis et al., 2010). We also used Breaks for Additive Seasonal and Trend function (bfast; Verbesselt et al., 2010) to detect abrupt changes in the time series trends (R software, Library Strucchange, and Package). The series of SAVI, lake area, and snow-ice cover of peatlands were tested for autocorrelations among quarters and years using ACF function in R software (Venables and Ripley, 2002; **Figure S1**).

To validate SAVI estimations with field measurements of vegetation cover recorded in the context of GLORIA network (Carilla et al., 2018a), we used Pearson correlations between vegetation cover measured in four summits and seasonal and annual SAVI calculated for each summit (with a buffer of 10m). We used percentage of vegetation cover in 1m2 quadrant (mean of 16 quadrants per summit, following field manual of Pauli et al., 2015) of four summits for the years 2006 (vegetation cover of summit 2 at 4240m), 2007 (cover of summits 1 at 4040 and 3 4450m), 2008 (cover of summit 4 at 4750m), 2012, 2017 and 2022 (cover of the four summits) (**Figure S2**).

#### 2.4.2 Relationships between the three indicators and climate records

To scan potential associations between selected indicators and climate records we performed correlation analyses. Acknowledging that vegetation and water balance results from the interaction between climate and local conditions we also evaluated the indicators that took into account spatial and seasonal variations. Vegetation productivity depends on seasonality and is likely to be controlled by water availability, which depends on climate and topography. Thus, we correlated the eight SAVI indicators, the five lake area indicators, and the three indicators of snow - ice cover of peatlands with the 18 climate variables. To evaluate possible delays in the response of vegetation productivity and water balance to climate signals we also assessed the correlation of all the indicators with climate records of the previous year.

To evaluate the synchronicity of the response of water balance to global and regional climate signals we explored the relationships between the remote sensed indicators of our study area and the average water surface of 15 lakes located along the Argentinean Puna (a system that is expected to have some similarities with Huaca Huasi).

We also analyzed the behavior of remote sensed indicators in response to El Niño and La Niña events. To attain a more rigorous statistical analysis of their relationship, taking into account the auto-correlation of time series, we used superposed epoch analysis (SEA; Prager and Hoenig, 1989; Rao et al., 2019), which compares the expected and observed values of response variables in a 6-yr window around ENSO year (the two previous and three subsequent years of each ENSO occurrence). SEA compares the observed mean values of SAVI/lake area/snow-ice cover of peatlands of these years with the complete time series by performing Monte Carlo simulations that randomly selects years, calculates expected means, and provides 95% CI, and hence identifies the statistical significance of the associations between 6 years and ENSO events of the years included in the time window.

## 3 Results

### 3.1 SAVI: Temporal characterization and relation with climate

**Figure 2:**
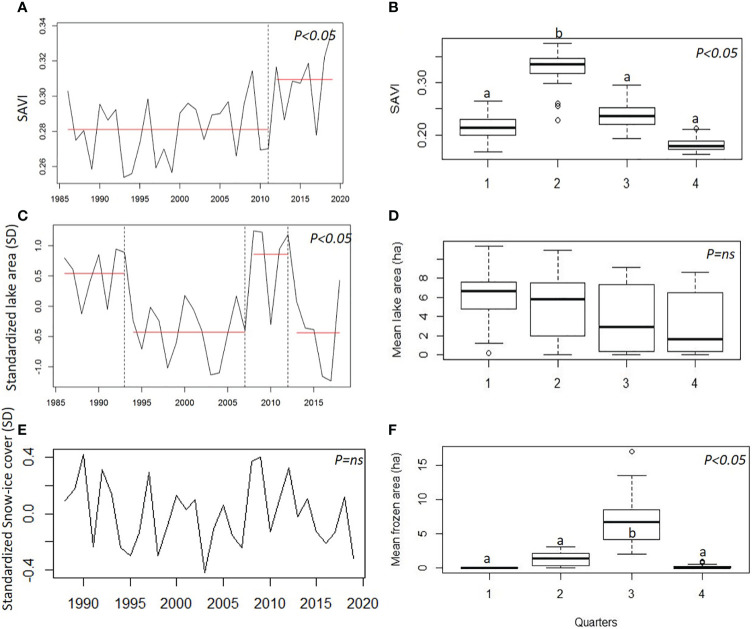
Temporal series of: **(A)** SAVI at annual scale, with periods of different average (red horizontal line), and **(B)** boxplot of mean SAVI for each quarter; seasonal effect; **(C)** Lake areas at annual scale with periods of different average (red horizontal line) and **(D)** boxplot of mean lake area for each quarter; seasonal effect; **(E)** Snow-ice cover of peatlands at annual scale and **(F)** boxplot of mean snow-ice cover for each quarter; seasonal effect. Different letters indicate significant statistical differences.

The annual average of SAVI showed high interannual variability, with the lowest values recorded in the first half of the time series (1989, 1993, 1994, 1997 and 1999). Peaks were observed every three to four years, with an overall significant increase in SAVI average values after 2011 (**Figure 2A**).

A seasonal effect was observed, with the highest values in autumn (quarter 2), and the lowest values in springtime (quarter 4; **Figure 2B**). The autocorrelation by quarters reflected the high seasonal effect and showed that vegetation of the same season was stable over time. The annual autocorrelation included positive association of one subsequent year (**Figure S1A**).

SAVI estimated for the three vegetation communities; peatland, plain and hillside showed similar patterns, with higher average in peatlands than in plains and hillslopes (33% higher on average).

Despite the low number of vegetation measurements, we found positive correlation between vegetation cover in GLORIA summits and seasonal and annual SAVI; (r=0.92, 0 = 88, 0 = 93, 0 = 87, p<0.05; for summer, autumn, winter and spring, respectively, and r=0.98, p<0.05 for annual SAVI; **Figure S2**).

**Figure 3:**
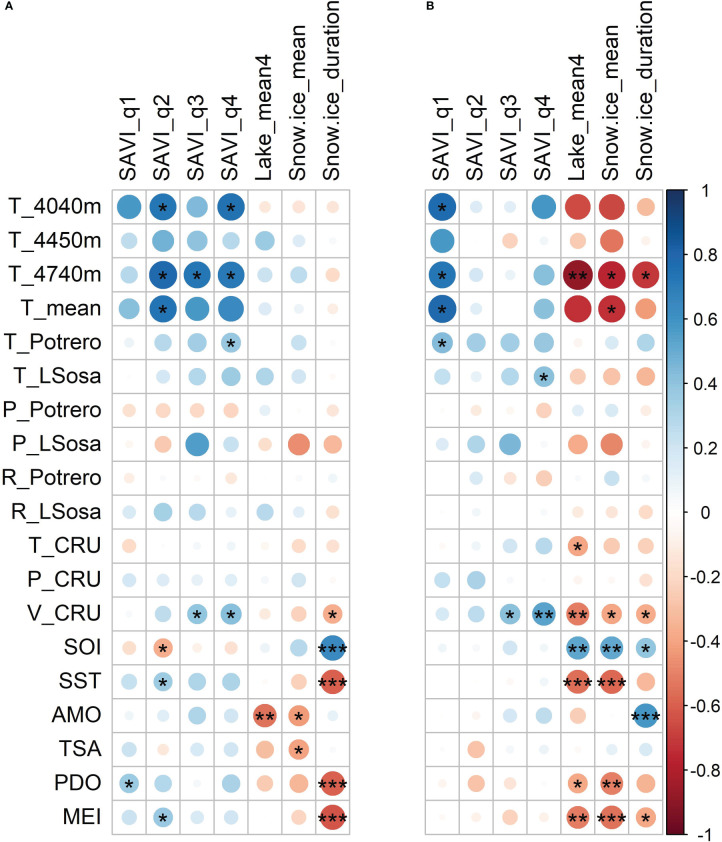
Heat maps showing the correlation between hydroclimate variables derived from remote sensing (vertical) and climate variables (horizontal). For the **(A)** current and **(B)** previous year (for climate codes see **Table 2**). ***p<0.01, **p<0.05, *p<0.1.

**Figure 4:**
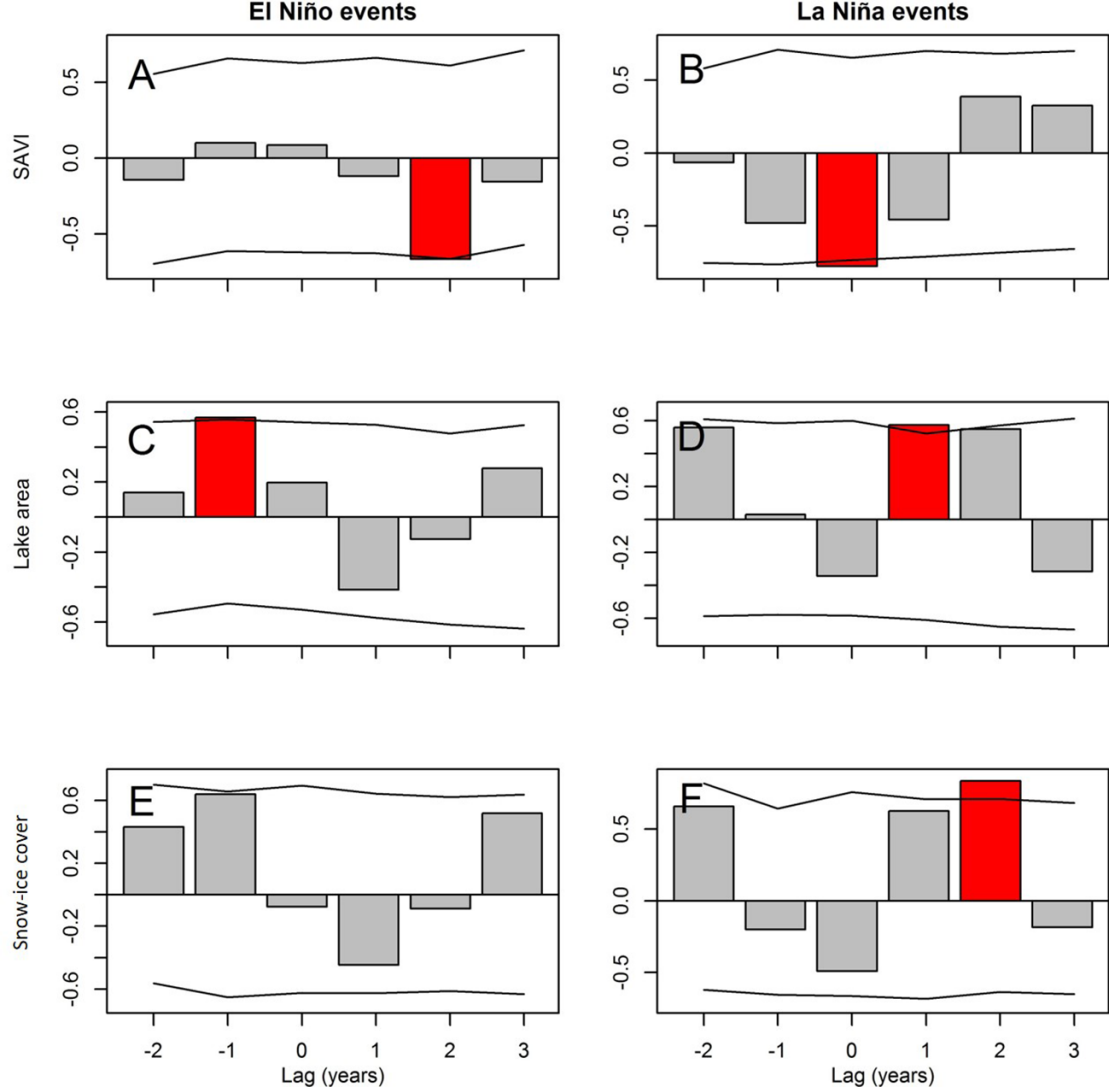
Relationships between the occurrence of El Niño and La Niña events (lag 0) over SAVI **(A, B)**, lake areas **(C, D)** and snow-ice cover **(E, F)** (standardized data). Red bars indicate statistical significance (p<0.05). Horizontal lines indicate 95% confidence intervals from the null model, derived from Superposed epoch analysis (SEA).

At local scale, SAVI was positively (marginally) correlated with soil temperature and air temperature records from meteorological stations (**Figure 3**). At the regional scale, SAVI presented positive correlations with vapor pressure (CRU database) for the current and previous year. On a global scale, SAVI presented positive correlation with SST and MEI and negative correlation with SOI (current year; **Figure 3**; **Figure S3**). During La Niña year, a decrease in SAVI was observed, as well as two years after El Niño event (SEA; **Figure 4**).

**Figure 5:**
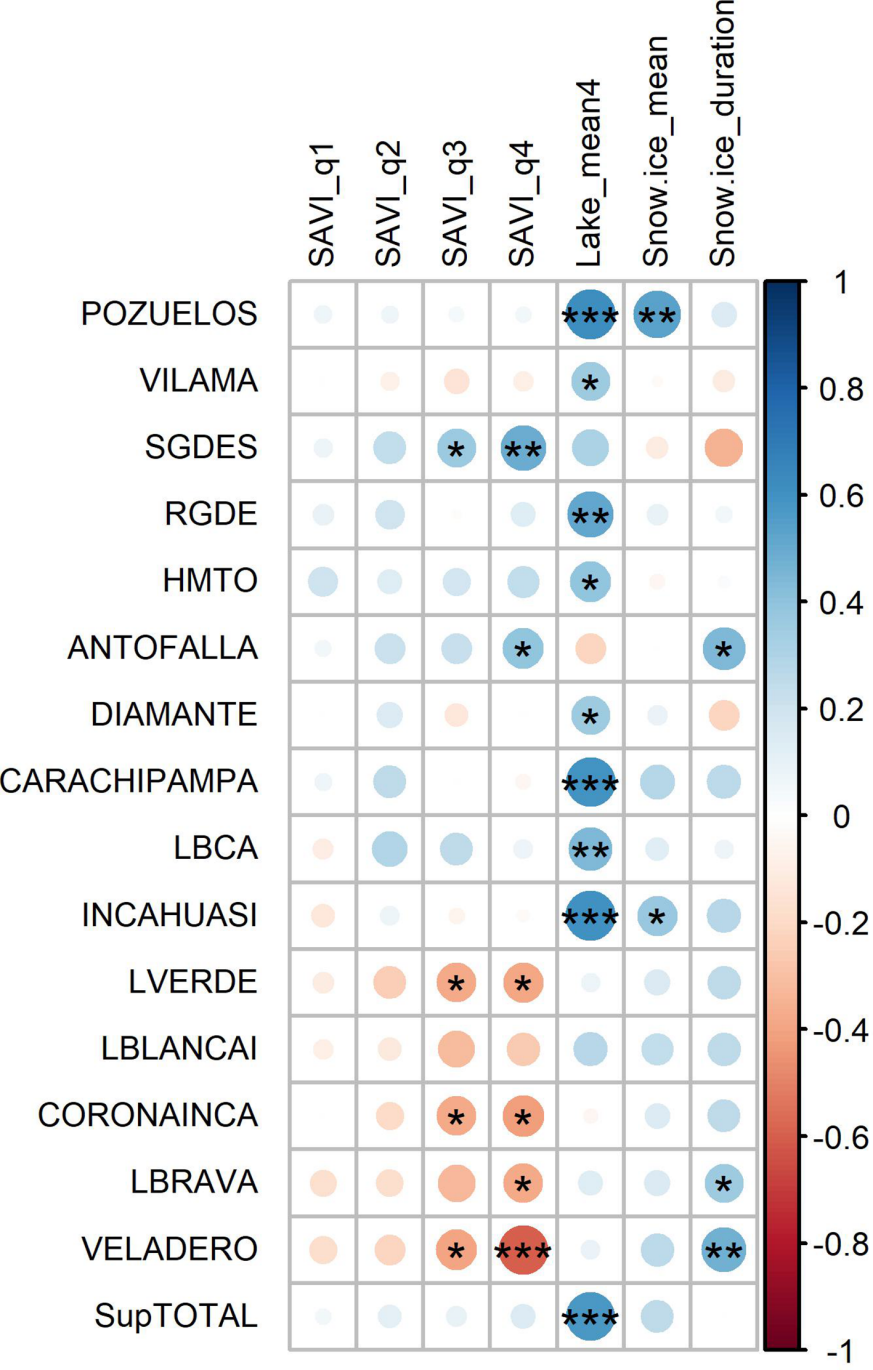
Heat map showing the correlations between the hydroclimate indicators derived from remote sensing (vertical) and 15 lakes from the Argentinean puna from Casagranda et al., 2019; horizontal). *** p<0.01, **p<0.05, *<0.1.

For the intrannual periods we found a positive significant correlation between SAVI and local soil temperature in autumn, winter and spring (quarters 2, 3 and 4), while this association faded in summer (quarter 1). Annual SAVI and autumn SAVI (q2) presented a similar relationship with climate variables (**Figure 3**). All the SAVI derived indicators showed weak correlations with lakes of the Puna region; the group of lakes with decreasing trend was negatively correlated with SAVI, and two lakes from the group with increasing trend were positively correlated (**Figure 5**).

### 3.2 Lake water areas: Temporal characterization and relation with climate

The four lake areas were positively intercorrelated (p<0.05; table 2), therefore, we used a single average value for the analysis. Stronger correlations were found between Grande, Amaicheña and Alta lakes (p<0.01; **Table 2**).

**Table 2:** Spearman correlations between Huaca Huasi lakes annual areas.

	Grande	Amaicheña	Alta
Amaicheña	0.72**		
Alta	0.86**	0.73**	
Escondida	0.27*	0.49**	0.26**

*p<0.05 **p<0.01.

Lake areas presented high interannual variability in the studied period, with variation coefficient, ranging from 39% in Escondida lake to 108% in Alta lake (**Table 3**). Mean water area for the four lakes was 4.3 ha (± 2.9), ranging from 0.1 (in 1998) to 8.9 ha (in 2008 and 2012), however, lakes did not dry up together, synchronously, in the same year.

**Table 3:** Lake water areas values; annual mean size and standard deviation (SD), range and coefficient of variation (standard deviation/mean *100).

Lakes	Mean size (SD) (ha)	Range: min - max (ha)	CV (%)
Grande (G)	7.6 (5.7)	0 - 18.9	75
Amaicheña (A)	8.7 (6)	0 - 15.3	67
Alta (Al)	0.6 (0.7)	0 - 2	108
Escondida (E)	0.5 (0.2)	0 - 0.7	40
Total average	4.3 (2.9)	0.1 - 8.9	67
Average G-A-Al	5.6 (3.8)	0.05 - 11.7	69

Along the 32 years, the mean lake area showed four periods with different means (**Figure 2C**). During the first period, from 1986 to 1993, lakes were larger than the mean (although in some years they almost dried up, e.g. 1988, 1991). In the second period, from 1994 to 2007, lakes were smaller than the mean. During a short period, from 2008 to 2012, lakes reached their greatest sizes (19.5 ha for Grande lake), with the two highest peaks of the series, 2008-2009 and 2011-2012. From 2013 to 2017, lake areas decreased and in 2018, the last year studied, lakes recovered their historical mean water area (**Figure 2C**). The bfast function identified a main break for the average of the four lakes in 2007, defining two periods with negative trends 1986 to 2007 (trend = -0.07) and 2008 to 2018 (trend = -0.16).

Seasonality of lake area series was less pronounced than SAVI’s (**Figure 2D**), and presented strong autocorrelation among quarters, which lasted for more than a year (**Figure S1B**).

Lakes did not vary significantly with seasons; therefore, we used a single annual value for correlation analyses with climate. On a local scale, average lake area presented negative correlation with soil temperature of the previous year. At regional scale, negative correlations with temperature and vapor pressure of the previous year were found (CRU database). On a global scale, lake areas showed strong positive correlation with SOI (previous year) and negative with SST, MEI, PDO (previous year), and AMO (current year; **Figure 3**; **Figure S3**).

ENSO was manifested in the lakes, with bigger lake areas a year after La Niña events (**Figure 4**). For Grande lake the variation was from 7.6 to 10.7 ha; for Amaicheña lake, from 8.6 to 13.1 ha; for Alta lake, from 0.6 to 0.9 ha; and for Escondida lake the effect was not significant (0.5 to 0.6 ha).

We found similar responses between the studied lakes and lakes along the Argentinean Puna; they presented different patterns of correlation with the same and the previous year. Nine lakes showed significant positive correlations (p<0.05) with the same year (Pozuelo, Vilama, Salinas Grandes, Rio Grande, Hombre Muerto, Diamante, Carachipampa, Laguna Blanca and Incahuasi), while the remaining six lakes were no significant correlated (**Figure 5**; **Figure S4**).

### 3.3 Snow-ice cover of peatlands: temporal characterization and relation with climate

The estimated area of snow-ice cover of peatlands varied from year to year with an average of 2.1 ha, ranging from 0.4 (2003) to 4.5 ha (1992; **Figure 2E**). The first month with snow-ice cover recorded was May (25% of the years) and the last month was October (59% of the years), with minimum and maximum duration from 1 to 6 months, and a mean duration of 4.5 months. No temporal trend was found (**Figure 2E**).

High seasonality was observed with the highest values in quarter 3, followed by quarter 2 (**Figure 2F**). No annual autocorrelation was observed (**Figure S1C**).

At a local scale, snow-ice cover of peatlands and its annual duration were negatively correlated with soil temperature of the previous year. At regional scale, snow-ice cover and duration were negative correlated with vapor pressure (CRU). At global scale, snow-ice cover and duration in peatlands showed positive correlation with SOI (previous year, and current year for snow-ice duration, as well). Snow-ice cover was negative correlated with AMO, TSA (current year), SST, PDO and MEI (previous year) and Snow-ice duration showed a positive correlation with SOI of the current year and AMO of the previous year (**Figure 3**, **Figure S3**). This positive/negative correlation with SOI/SST, was also manifested with more extensive snow-ice cover of peatland two years after La Niña events (2 vs 3 ha; **Figure 4**), and lower cover during El Niño events (**Figure 4**).

Snow-ice cover of peatlands and its duration were positively correlated with two and three, respectively different puna lake areas (**Figure 5**).

### 3.4 Climate variables relationships and trends

Temperature data at local and regional scales were positively correlated, also with vapor pressure (r = 0.7 and 0.6, respectively, p < 0.01), and weakly negatively correlated with local precipitation (r = -0.56, p = 0.09). Both river gauges were positively correlated with regional precipitation (r=0.58 and 0.61, p<0.01) and negatively with regional temperature (r=0.5, p< 0.01; CRU database). On a global scale, SST and MEI were strongly positively correlated with local (r=0.8 p<0.01 both) and regional (only SST, r=0.4, p=0.02) temperature, while SOI was strongly negatively correlated with local temperature (r= -0.8, r=0.003) and weakly positively with precipitation (**Figure S5**). Local temperature presented positive trends over time (R^2 =^ 0.15 and R^2 =^ 0.2, p<0.04), as vapor pressure (CRU regional data; R^2 =^ 0.1, p=0.07), AMO and TSA (global variables; R^2 =^ 0.5 and R^2 =^ 0.1, p<0.04), while local precipitation presented a negative trend over time (R^2 =^ 0.11, p=0.04).

### 3.5 SAVI, lakes and snow-ice cover relationships

**Figure 6:**
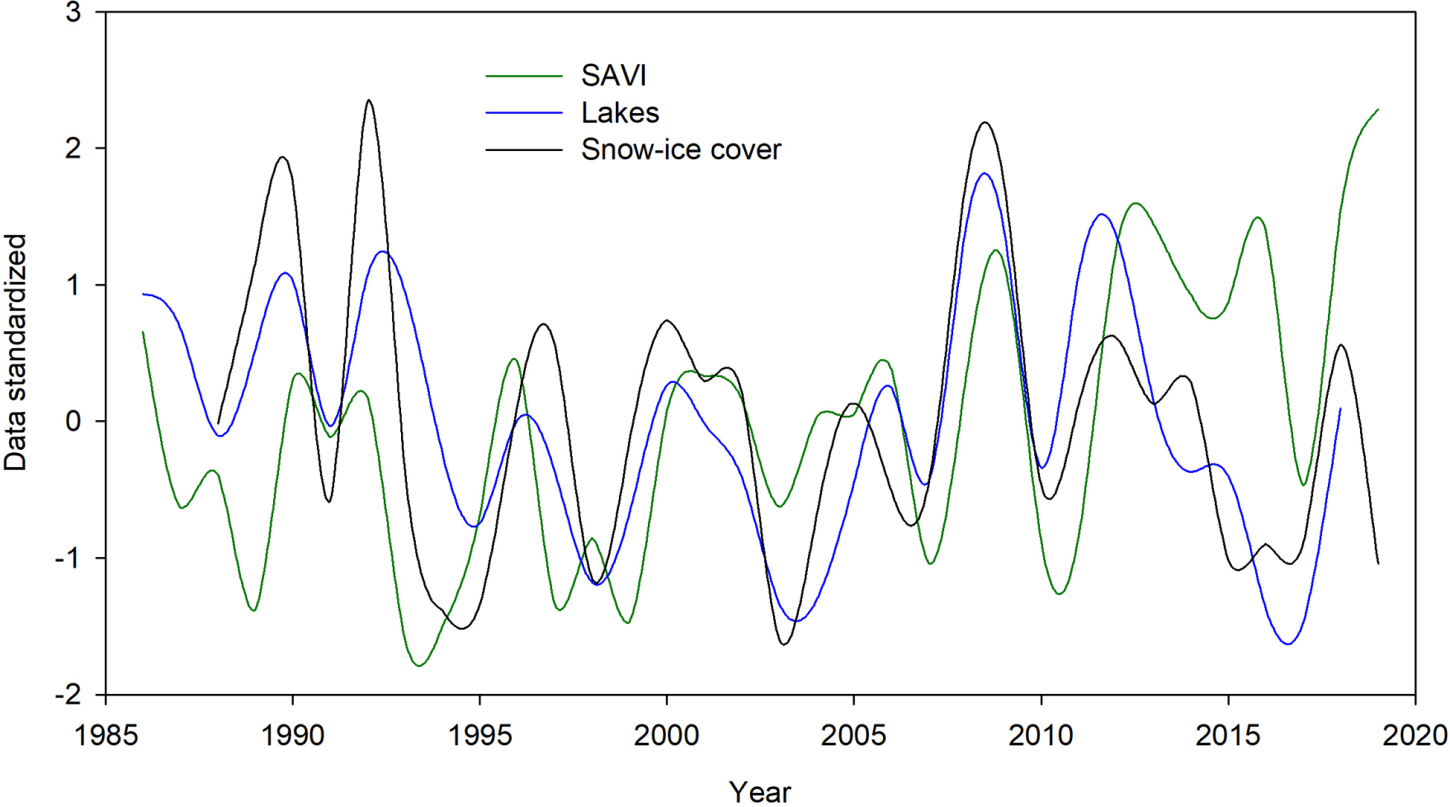
Trends in annual SAVI, lake areas and snow-ice cover of peatlands in the last 32 years (Pearson’s correlation for the current year: SAVI and lake areas r = ns, SAVI and snow-ice, r = ns, Lakes and snow-ice r = 0.76, p<0.05).

The cross correlation between SAVI and lake areas showed a positive association between the complete series in the current and subsequent quarters (**Figure S7**), with SAVI seasonal fluctuations more pronounced than in lakes, as observed in **Figures 2B, D**. However, the annual series of SAVI and lake area were negatively correlated with a year of delay (r=-0.33, p<0.05; **Figure 6**), this difference was more pronounced after 2011, since both series showed opposite trends; increasing in SAVI values and decreasing in lake areas, with a common valley in 2017 of low values (**Figure 6**; **Figure S6**).

The cross correlation between SAVI and snow-ice cover in peatlands for the complete series showed a strong negative association for the 32 years, reflecting the opposite patterns in seasonality of both series, observed in **Figures 2B, F**. Extracting the seasonality effect, i.e. at annual scale, no significant correlation was observed between SAVI and snow-ice cover nor its annual duration (**Figure 6**; **Figures S6, S7**).

On the other hand, the cross-correlation between lake areas and snow-ice cover in peatlands showed a positive correlation between the complete series for the subsequent quarters (**Figure S7A**). For the annual series, lake area and snow-ice cover in peatlands were positively correlated for the current and the subsequent year (r= 0.76 and 0.77, respectively, p<0.05; **Figure 6**; **Figure S7B**), as well as lake area and the annual duration of the snow-ice cover for the same and previous year (r=0.5, 0.47, respectively, p<0.05).

From the association of the three variables at quarter scales, we observed an effect over the subsequent season (i.e. with a season of delay) between most variables: autumn SAVI (q2) and winter lake area (q3) were negatively correlated (r=-0.4, p=0.02). Snow-ice cover of peatland of autumn/winter/spring (q 2/3/4) were positively correlated with lake areas in winter (q3, r=0.33, p<0.07)/spring (q4, r=0.68, p=0.001)/summer of the subsequent year (q1, r= 0.38, p=0.04), respectively. Winter snow-ice cover of peatland (q 3) was positively correlated with spring SAVI (q4, r=0.49, p<0.05).

## 4 Discussion

The combination of high spatial and temporal resolution hydroclimate indicators derived from remote sensing for studying high Andean ecosystem dynamics allows us to depict the functioning of high Andean ecosystems in response to climate variability over the last three decades. Especially, considering the vulnerability of the dry Central Andes to climate change and their central role in the global economy (Izquierdo et al., 2015a). We highlight the potential of SAVI, the area of lakes and snow-ice cover in peatlands as indicators of ecosystem’s responses to climate at different scales. The information provided by these indicators is complementary; vegetation productivity seems to depend on temperature, lake area mainly reflects the water balance of previous years and snow-ice cover is associated with available surface water during the dry winter season. Overall, we observed a temporal trend of warming that fostered an increase in vegetation activity but there were no clear signs of decreasing in water balance, which was highly variable.

Our results highlight the usefulness of SAVI to generate spatially explicit time series of photosynthetic activity in high Andean ecosystems with good spatial resolution. Our study showed that SAVI reflects field measurements of vegetation cover, and the increment trend in the last years resulted in concordance with an increment in plant cover, associated with higher climate variability (Carilla et al., 2018). Besides, the SAVI time series constitute long-term indicators of ecosystem functioning, and are especially useful to complement scarce field instrumental records. Although this type of information can hardly suggest the mechanisms of vegetation change (Gaitán et al., 2015), its use has been touted in conservation, to describe the state of ecosystems affected by human activities, including fire, grazing, etc. (Yang and Guo, 2011; Arnett et al., 2015; Ilhom, 2016). So, although climate is likely only one amongst multiple processes affecting SAVI, the combination with additional indicators, such as lake area, reinforce the relation to climate variability (Carilla et al., 2013; Zhang et al., 2019).

Lake area as a regional indicator of water balance permits reconstructing a time series of available water, which results from the inflows by precipitation modulated by global forces and outflows by evapotranspiration (Stephenson, 1998), mainly affected by local temperature. However, the use of lake areas as a water balance indicators restricted to a limited range of conditions. Sometimes shallow lakes may dry up or they may overflow (when they are saturated) (Dangles et al., 2017). In this study, we observed a restricted temporal window of lake area, plenty of dry-full cycles showing a recovery from very low values reported at the turn of the 21st century (Halloy, 2002). However, the maximum possible values of some of these lakes have not been systematically recorded (e.g. Laguna Grande never reached its maximum within, at least, the last century and it can get a lot larger; Halloy, 2002). The averaging of the four lakes smooths the signal of individual lakes, increasing the sensitivity range of the record.

The influence of climate over vegetation, lakes and snow-ice, at multiple scales, highlights their power as hydroclimate indicators and important metrics of ecosystem functioning. In this study, we emphasize the integral interpretation of the indicators and the ability of their spatialization and their relations with climate, studied for vegetation and lakes (Carilla et al., 2013; Morales et al., 2015; Zhang et al., 2019; Bianchi et al., 2020; Santamans et al., 2021), and for snow-ice cover (Brown and Duguay, 2010; Garcia Gonzalez and Otto, 2015).

Although a negative correlation was observed between SAVI and lake areas, this decoupling relationship was mainly driven by the last years, when SAVI and lakes/snow-ice series tended to diverge. Despite this divergent pattern, the feedback between snow-ice cover, SAVI and lakes is quite informative. This study shows how vegetation and lake areas in spring, the season with the highest water deficit, are both favored by the winter snow (Otto et al., 2011). However, a negative feedback is observed between vegetation and lake areas at the end of the growing season that may have implications under a global warming context, e.g. more productive vegetation but at the same time more water-demanding and more evapotranspiration, and hence less water reserves downstream, as observed in other semi- arid ecosystems (Feng et al., 2016). It is also likely that the water that reaches the peatlands and is not evaporated feeds the associated lakes, as well as the water that falls in hillslopes and plains during winter feeds peatlands in the driest season and at the beginning of the rainy growing season through underground flows (Otto et al., 2011).

The response of vegetation to temperature, also observed by other authors (Maggi et al., 2020) is probably associated with a thermic limit to plant growth described by global analyses of montane systems (Körner, 1999; Pauli & Halloy, 2019). Likewise, the increasing trend in SAVI in the last years, also observed in other high mountains (Gaitán et al., 2015; Chávez et al., 2019; Campbell et al., 2021; Anderson et al., 2021), could be a response to the increase in temperatures (in tune with global warming, Xu et al., 2019; Xu et al., 2020) recorded in both lower elevation meteorological stations. Other authors have suggested that increasing vegetation activity in montane systems is related with the occurrence of anomalous rain and snow episodes (Otto et al., 2011; Anderson et al., 2021). Despite our lack of long-term instrumental data, measurements of soil temperature indicate an upward temperature trend (even the short series), which is supported by soil measurements since 1970 for the area data from Halloy (2002, and more recently data not published), accurately reflecting global warming trends (Vuille et al., 2018; Tovar et al., 2022). Soil temperature increases are especially indicative of long term warming because deep soil temperature is more stable than surface temperature, and therefore long-term signals can be better distinguished from background variability. At the same time, soil temperature is more sensitive to vegetation cover than air temperature, conditioning biological and biogeochemical processes (Yu et al., 2022). In the contrary, precipitation patterns present high spatial variability due to topography (variation in wind, albedo, heat flux; Houze, 2012), making difficult modelling and forecasting analysis.

The lagged association of lake area with water balance (global scale precipitation and local temperature) suggests the presence of intermediate processes that delay the response to climate. The hydrological behavior of these lakes is a complex result of precipitation and evaporation. However, vegetation (through evapotranspiration), infiltration, soil types, and subsoil geology (which influence lag times, and subterranean losses) may also influence the system (Hutchinson, 1967). Despite this complexity, the synchronicity of fluctuating water areas with lakes along the Puna (Casagranda et al., 2019), shows that these indicators reflect regional weather patterns. The effect of the Pacific Ocean over this part of the subtropical Andes seems to be stronger than the Atlantic effect; manifested through stronger responses mainly hydrology to SOI, SST, PDO and MEI, than to AMO and TSA, but also with opposite effects over vegetation (e.g. positive correlation with SST, PDO and MEI over SAVI; **Figure 3**). The influence of ENSO on the three indicators is a manifestation of this Pacific effect. Besides, during La Niña years, SAVI showed lower values and lakes presented larger areas probably because La Niña is associated with lower SST (26.5°C vs 27.7°C in El Niño) and PDO, and higher values of SOI, which is associated with precipitation (Ropelewski and Halpert, 1989; Karabörk and Kahya, 2007). The opposite pattern is observed for El Niño years, when SAVI also presented lower values (with two years of delay), as other authors observed for the Andes (Maggi et al., 2020). The effect of precipitation decreasing and temperature increasing trends, over freshwater availability and vegetation growth, was observed in previous studies in the area, where higher plant community turnover and increase in vegetation cover and species richness were observed over a decade, suggesting a response to short-term climate variability associated with ENSO (Carilla et al., 2018).

Despite the common behavior between lakes, and similar responses to climate, each lake also exhibits its own dynamics, observed in the absence of years when all lakes dry up, or to an extent where there is only a bit of muddy water. However, there are few seasons when all the lakes dry up (autumn and summer in 2016, and autumn and winter in 2017). Because of its particular dynamics, the only lake that has not dried up is Escondida, although its area decreased substantially. In some way, this reflects the complexity of these systems, indicating that each particular lake responds to local factors such as the groundwater availability, the depth of the lake and the sub-basin topography (Dangles et al., 2017; Casagranda et al., 2019). Grande Lake encompasses a group of several lakes of different sizes, located in the same sub-basin as Alta Lake, draining east toward montane forests and the Chaco lowlands. The Amaicheña Lake, located north of the study area, drains toward the dry inter-Andean valleys to the west. The most isolated and different system is the Escondida lake, which is deeper and smaller, and is located in a small, deep and diverse gorge (habitat of condor, puma, big rodents, etc) and is one of the few places in this mountain range with rock glaciers (Halloy, com pers, Ahumada et al., 2017). The first insights of Cumbres Calchaquíes are some notes and photographs that allow a visualization of lake areas at some points back to the 1890s. During the last century, Halloy (2002) observed the highest records in lake areas in 1972. After the 70’s this group of lakes seems to be affected by drought conditions, evidenced in a generalized decrease in lake areas, with some humid peaks (e.g. in 2012), and some years with lakes mostly dry (e.g. in 2017), even those lakes that were previously permanent (Halloy, 2002; Casagranda, 2010).

The complex topography and scarce instrumental data on mountains of South America hinder studies of ecosystems functioning and their relation to climate change. The use of new tools, such as remote sensed indicators, which turns mandatory in this study present some constraints. Ground-thruthing with permanent plots, which encompass smaller temporal and spatial scales, is also necessary to explain the underlying causes of observed changes (Dangles et al., 2017; de Bello et al., 2020). The GLORIA initiative (Pauli et al., 2015) with plots in Cumbres Calchaquíes since 2006 (Carilla et al., 2018), contribute to such ground-truthing with globally standardized methods, however has the limitation of real time resolution and in some cases observer bias (Kapfer et al., 2017). Thus, our challenge is to fine-tune the methods, to combine both tools, field work and remote sensing, for obtaining a more complete picture of the ecosystem dynamics and to develop long-term studies (Halloy, 2002).

Long-term monitoring of high mountain hydrology allows understanding and anticipating future ecosystem scenarios in response to climate change, which is relevant when considering the importance of mountains as water towers supporting human life and biodiversity (Immerzeel et al., 2020). In Cumbres Calchaquíes, superficial (peatlands and rivers) and underground water are the main freshwater reservoirs and are the main suppliers of this resource to the western dry valleys (with more than 5000 inhabitants). In a context of a densely populated province (Tucuman province; 22.500 km^2^ with 1.5 million inhabitants) with an increasing anthropic pressure over mountains, where important rivers are born (García et al., 2017), this study is relevant for water management. We highlight the potentiality to generate a systematic accessible replicable tool for monitoring eco-hydrology above 4000 m asl, in subtropical mountains.

## 5 Conclusion

The complex topography and scarce instrumental data on mountains of South America make it difficult to develop long-term studies of ecosystems in relation to climate change. We highlight the potential of SAVI, lake areas and snow-ice cover in peatlands as indicators of ecosystem response to climate at different scales, by providing complementary information. We found a positive trend of SAVI over time, probably associated with increasing temperatures. Vegetation productivity increases in warmer years and the lake area reflects the water balance of previous years. In this system, the snow-ice cover is associated with available surface water, strongly correlated with lake area. Global scale atmospheric phenomena influence vegetation and hydrology in these mountains; SAVI presents lower values during La Niña year and two years after El Niño, while lake area and snow-ice cover are higher after La Niña event. Also reflected through the effect of SOI, SST, PDO and MEI. This study provides a systematic accessible replicable tool for long-term monitoring eco-hydrology in subtropical mountains above 4000 m asl. The dry Puna, stretching roughly from 18°S to 28°S, encloses ca. 300.000 km2 of highlands including the “Lithium Triangle” with little or no meteorological records. Our methodology may be useful in a region with critical economic and socio-political values. Not just for the countries involved but for the whole world.

## Data availability statement

The original contributions presented in the study are included in the article/**Supplementary Material**. Further inquiries can be directed to the corresponding authors.

## Author contributions 

JC conceived of the study. JC and EA drafted the manuscript. EA analyzed data. JF and EC prepared the data set. All authors contributed to the article and approved the submitted version.
