# Supplementary material for: Hydroclimate and vegetation variability of high Andean ecosystems
Source: Front Plant Sci. 2023 Jan 20;13:1067096. doi: 10.3389/fpls.2022.1067096 (PMC9895849; doi:10.3389/fpls.2022.1067096)
Supplement: Supplementary file 1 [file DataSheet_1.docx]

# Supplementary Material

## Supplementary Figures and Tables

A)
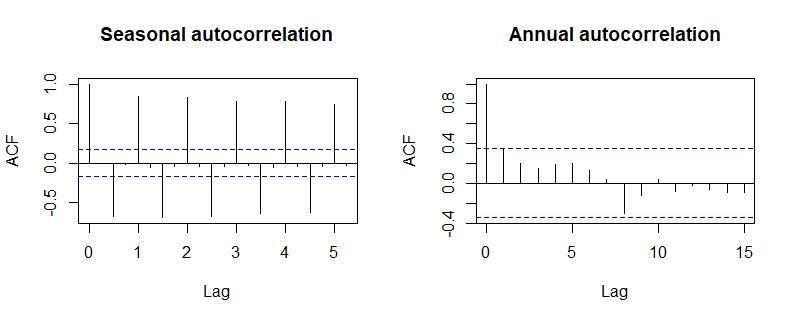


B)
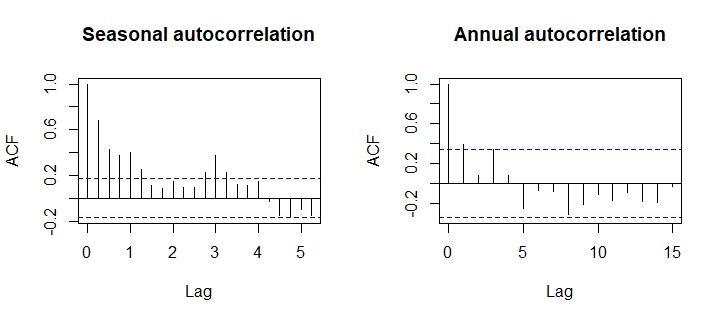


C)
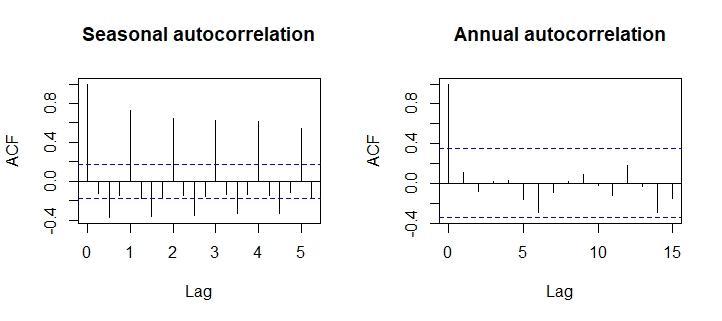


Figure S1. Autocorrelation among quarters (lag= quarters), and years (lag= years) of A) SAVI (over 4000 masl), B) mean lake areas (average of the four lakes) and C) snow-ice cover of peatlands. Dashed lines indicate a 95% confidence interval.


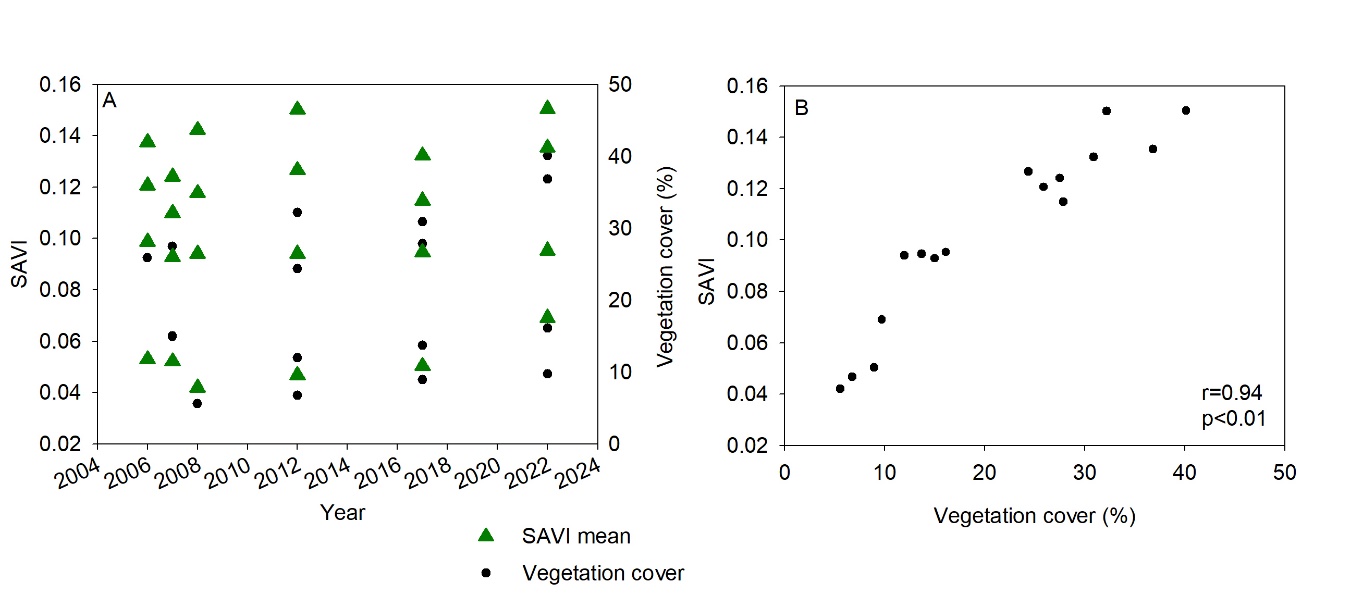


Figure S2 A) temporal trends in vegetation cover and SAVI (calculated for the four summit) B) scatterplot of both variables (for the common period 2006 to 2022).


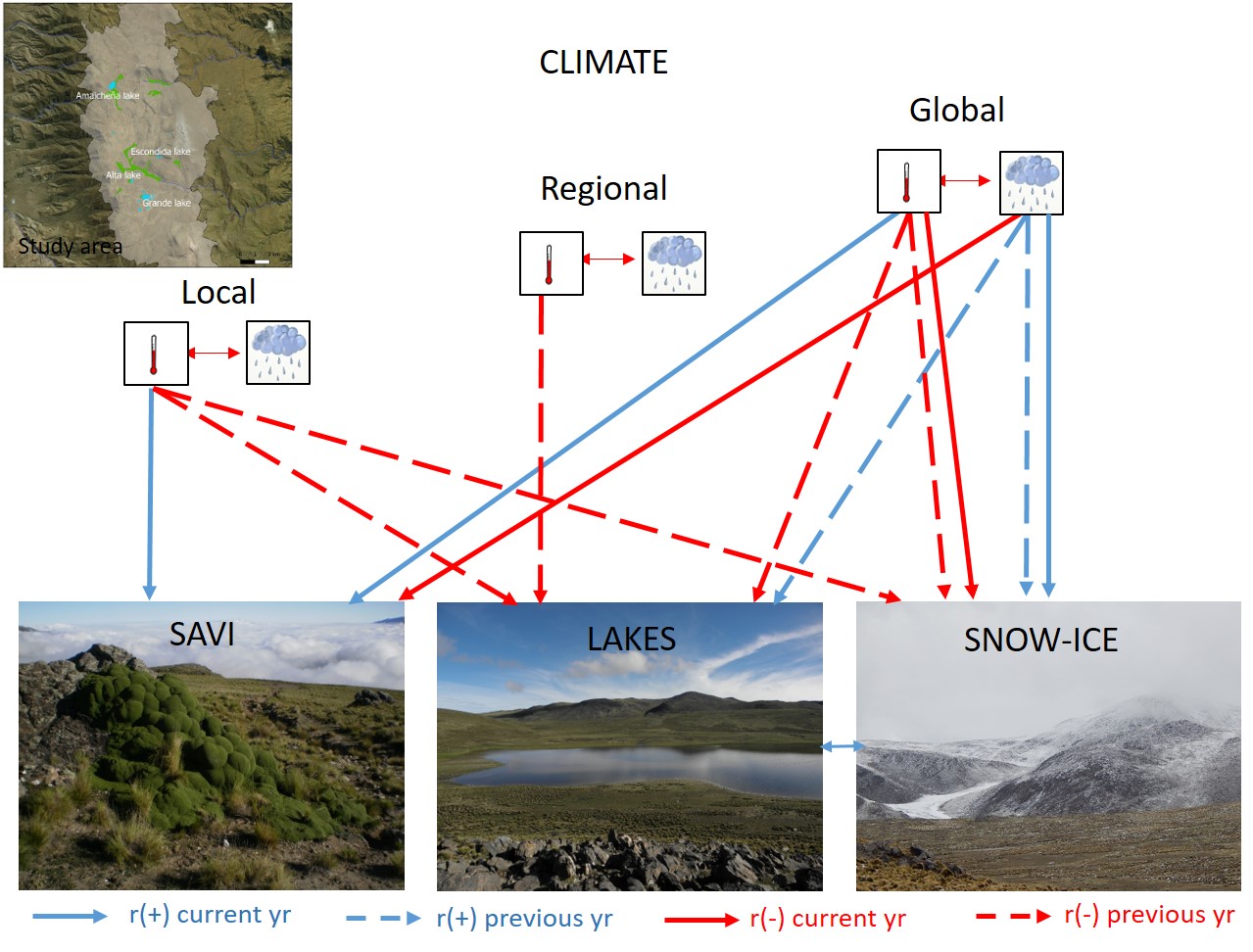


Figure S3. Graphical abstract of the main relationships between the three ecohydrological indicators and climate variables at local, regional and global scales, for the current and previous years in the study area (map at the upper corner; grey polygon indicate area over 4000 m asl). Red / blue lines indicate negative/ positive significant (Pearson) correlations for the current (continuous line) and previous (dashed line) years. Photos are representative of the vegetation, lake areas and snow-ice cover indicators.


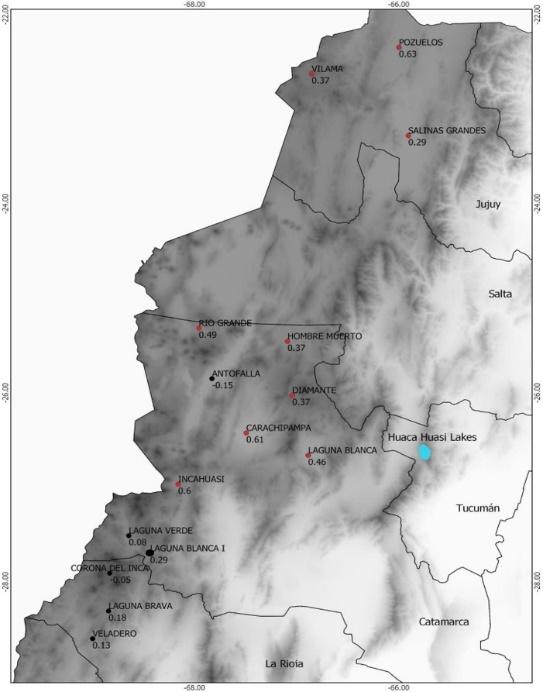


Figure S4. Lakes distribution along the Argentinean Puna region and Huaca Huasi lakes (light blue). Red points represent positive significant correlations and black points non-significant correlations, p<0.05, (between puna and Huaca Huasi lakes in the current year. Common period 1986 – 2017).


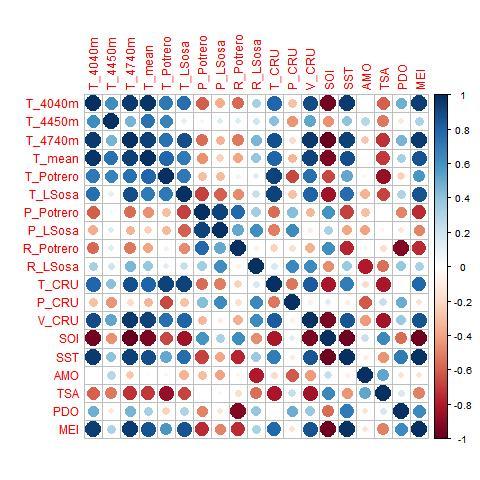


Figure S5: Relationships among climate variables for the same year. Color circles correspond to the correlation coefficient scale. Climate codes: see table 2


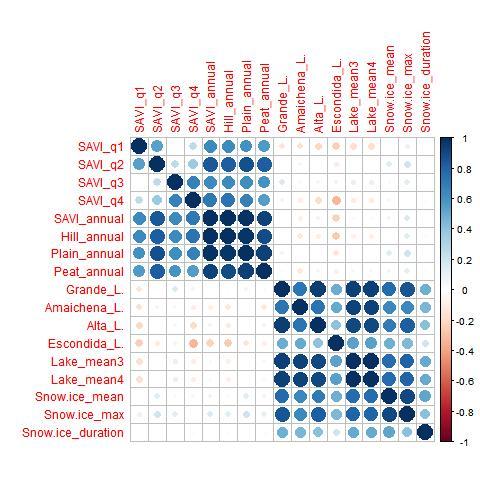


Figure S6. Relationships among indicators for the current year. Color circles correspond to the correlation coefficient scale. indicators are values of SAVI for summer (q1), autumn (q2), winter (q3), spring (q4), annual SAVI, SAVI for hillsides, for plains, and for peatlands. Lake areas for Grande, Amaichena, Alta and Escondida lake, mean lake area for the four lakes and disregarding Escondida. Mean, max and duration of snow-ice cover of peatlands.

A)


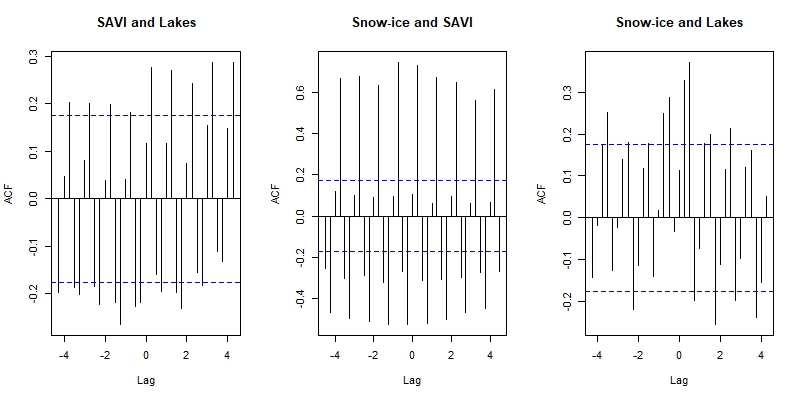


B)


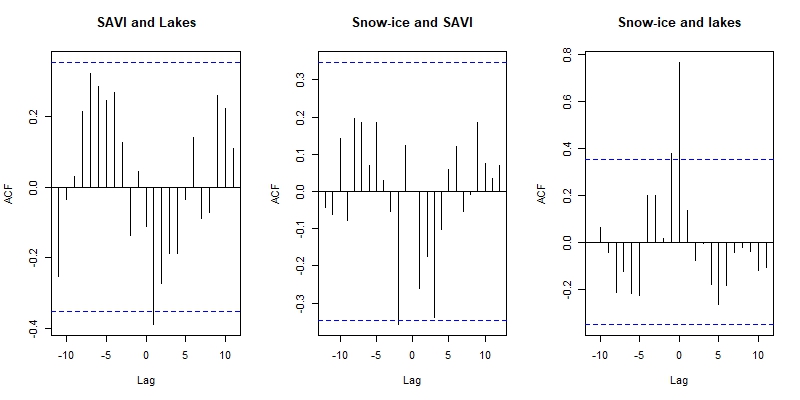


Figure S7. Cross correlograms between SAVI, mean lake areas and snow-ice cover of peatlands A) for the complete series (lag = quarter), B) for the annual series (lag= year). Dashed lines indicate 95% confidence interval.
